# Neck Circumference Is Associated With Poor Outcome in Patients With Spontaneous Intracerebral Hemorrhage

**DOI:** 10.3389/fneur.2020.622476

**Published:** 2021-02-01

**Authors:** Yujian Li, Huiqing Zhou, Xiang Yang, Jun Zheng, Fan Zhang, Mangmang Xu, Hao Li

**Affiliations:** ^1^Department of Neurosurgery, West China Hospital, Sichuan University, Chengdu, China; ^2^Department of Intensive Care Unit, Fourth People's Hospital of Sichuan Province, Chengdu, China; ^3^Department of Neurology, West China Hospital, Sichuan University, Chengdu, China

**Keywords:** prognosis, predictor, obesity, neck circumference, intracerebal hemorrhage

## Abstract

**Objective:** This study aimed to assess the association between neck circumference (NC) and functional outcome in intracerebral hemorrhage (ICH) patients.

**Methods:** We prospectively analyzed data from ICH patients who received treatment at our institution from January 2018 to November 2019. Patients were categorized into two groups according to 180-day modified Rankin scale (MRS) scores. Univariate and multivariate analyses were performed to assess whether NC was associated with poor outcome in ICH patients. Receiver operating characteristic (ROC) curve analysis was performed to determine the significance of NC in predicting the functional outcome of ICH patients.

**Results:** A total of 312 patients were enrolled in our study. Multivariate logistic regression analysis indicated that NC was an independent predictor of poor 180-day functional outcome [odds ratio (OR) = 1.205, 95% confidence interval (CI): 1.075–1.350, *p* = 0.001]. ROC analysis revealed that NC could predict poor functional outcome at 6 months.

**Conclusions:** NC is an independent predictor of unfavorable functional outcome at 6 months in ICH patients.

## Introduction

Spontaneous intracerebral hemorrhage (ICH) is a devastating health event accounting for 10–15% of all strokes (1, 2) and has characteristics of high mortality and morbidity and limited treatment options (3). Obesity is one of the major risk factors for stroke (4) and is associated with increased morbidity and mortality in the general population (5, 6). However, obesity appears to confer a survival advantage in patients with certain diseases, including heart failure, coronary artery disease, and chronic kidney disease (7–9). In addition, recent studies have linked this phenomenon, known as the “obesity paradox,” to ICH (10, 11).

Body mass index (BMI) is an indicator that is most commonly used to assess obesity. However, previous studies have shown that neck circumference (NC) could be used as a simple, feasible, and stable evaluation index because it is not easily affected by eating or body position (12, 13). In addition, NC is related to oropharyngeal fat infiltration, which narrows the upper respiratory tract (14). However, little is known about the role of NC in patients with ICH. The purpose of this study was to explore the relationship between NC and the prognosis of ICH.

## Materials and Methods

### Patients

We defined the inclusion criteria as follows: (1) a diagnosis of intracranial hemorrhage using computed tomography (CT), (2) routine blood examination and laboratory tests conducted within 24 h after admission, and (3) age ≥ 18 years. Patients who met the following criteria were excluded: (1) ICH attributable to aneurysm, arteriovenous malformation, or Moyamoya disease; (2) ICH attributable to acute cerebral infarction or thrombolysis of cerebral or myocardial infarction; (3) prior systemic diseases, such as immunological disease, neurological disease, recent infectious disease, severe hepatic or renal dysfunction, and coagulation dysfunction; (4) patients with isolated intraventricular hemorrhage (IVH); and (5) patients with a history of neck surgery. Brain CT angiography (CTA) or magnetic resonance angiography (MRA) was used to exclude aneurysm, arteriovenous malformation, or Moyamoya disease.

### Clinical and Laboratory Parameters

Baseline clinical and demographic parameters, including NC, BMI, height-to-NC ratio (HNR), age, sex, medical history, Glasgow Coma Scale (GCS) score on admission, blood pressure, cigarette and alcohol use, history of stroke, medical history of hypertension, diabetes mellitus, and history of endotracheal intubation and tracheotomy, were collected at hospital arrival.

**Figure 1:**
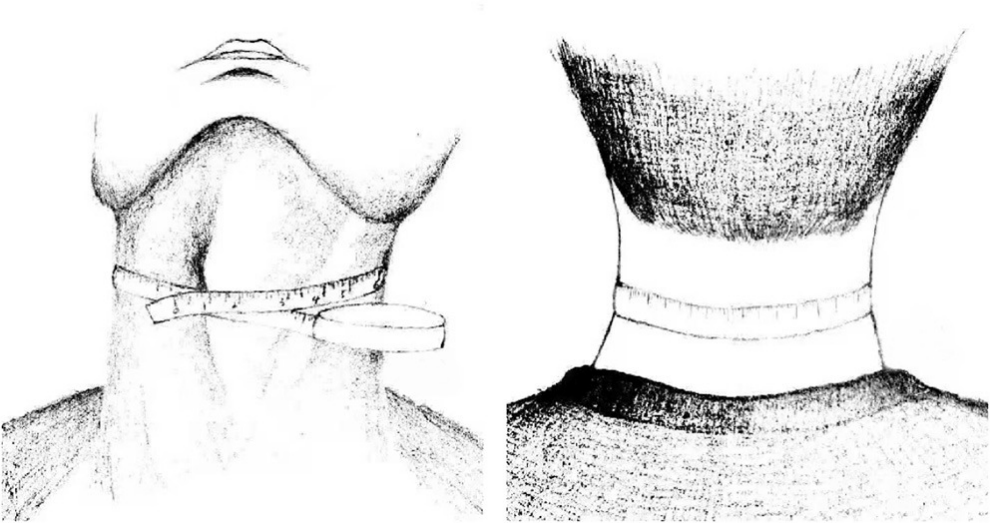
Schematic diagram of the measurement method of NC.

Laboratorial variables, including white blood cells (WBCs), platelets, blood glucose, cholesterol, high-density lipoprotein cholesterol (HDL-C), and low-density lipoprotein cholesterol (LDL-C), were also recorded. NC (cm) was measured to the nearest 1 mm with plastic tape. The tape was placed as horizontally as possible, at a point below the larynx (thyroid cartilage) and perpendicular to the long axis of the neck (keep the tape line on the front of the neck at the same height as the tape line on the back of the neck, Figure 1). Two reviewers independently estimated the values of NC. Any disagreement between the two reviewers was resolved by consensus. Height (m) and weight (kg) were measured at the bedside by trained staff on admission using a tape measure and a digital weight scale, respectively, and BMI was calculated according to the standard formula (kg/m^2^). Admission HNR was calculated as the ratio of the height to the NC.

Radiological results collected from head CT within 24 h after admission to the hospital included hematoma location, hematoma size, and the presence of IVH. Hematoma volume was measured by the ABC/2 method as described previously (15). Two reviewers independently estimated all the head CT scans. Any disagreement between the two reviewers was resolved by consensus.

The functional outcome was assessed according to the 180-day modified Rankin scale (MRS) score obtained by telephone or during outpatient visits. An MRS score ≥3 was defined as an unfavorable outcome, including severe disability, persistent vegetative state, and death.

### Statistical Analysis

All baseline characteristics, including clinical variables, laboratory parameters, and radiological data, were compared between patients with poor outcomes and those with favorable outcomes. Continuous variables are expressed as mean ± standard deviation or median with interquartile range (IQR) for normally distributed and non-normally distributed variables, respectively, whereas categorical variables are expressed as frequency and percentage. Univariate analyses were conducted by independent *t*-test, Mann–Whitney *U*-test, chi-square (χ^2^) test, or Fisher's exact test. Independent *t*-tests or Mann–Whitney *U*-tests were applied to compare continuous variables. The chi-square (χ^2^) test or Fisher's exact test was used to compare categorical data. Variables significant at the *p* < 0.20 level in the univariate analysis were retained in the multivariate model. For interpretation purposes, some variables were classed as follows: GCS score as “13–15 points,” “9–12 points,” and “3–8 points”; and hematoma location as “lobe,” “basal ganglia,” “thalamus,” “cerebellum,” and “brainstem.” Receiver operating characteristic (ROC) analysis was performed to indicate the predictive value of NC for the functional outcome of ICH patients. The cut-off value of NC was decided by the Youden index from the ROC curve, and sensitivity and specificity were used to calculate the Youden index (sensitivity + specificity – 1). A value of *p* < 0.05 was considered statistically significant. All the above-mentioned statistical analyses were performed by SPSS version 21.0 (SPSS, Chicago, IL, USA).

## Result

**Figure 2:**
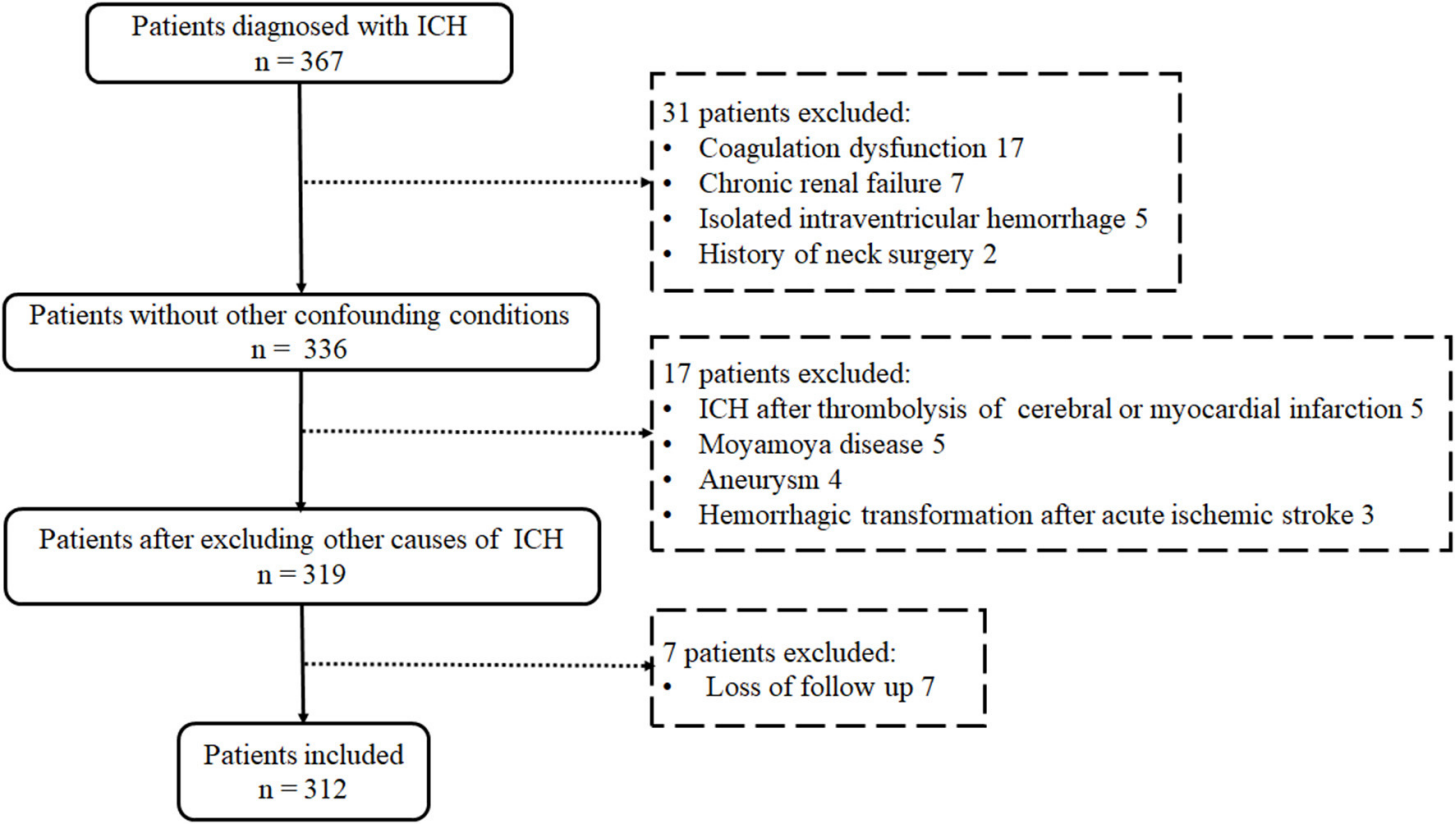
Flowchart of study enrolment.

From January 2018 to November 2019, 312 consecutive patients (229 males and 83 females) with spontaneous ICH who met the inclusion criteria were enrolled in this prospective study, and the rate of loss to follow-up was 2% (7/319, Figure 2).

**Figure 3:**
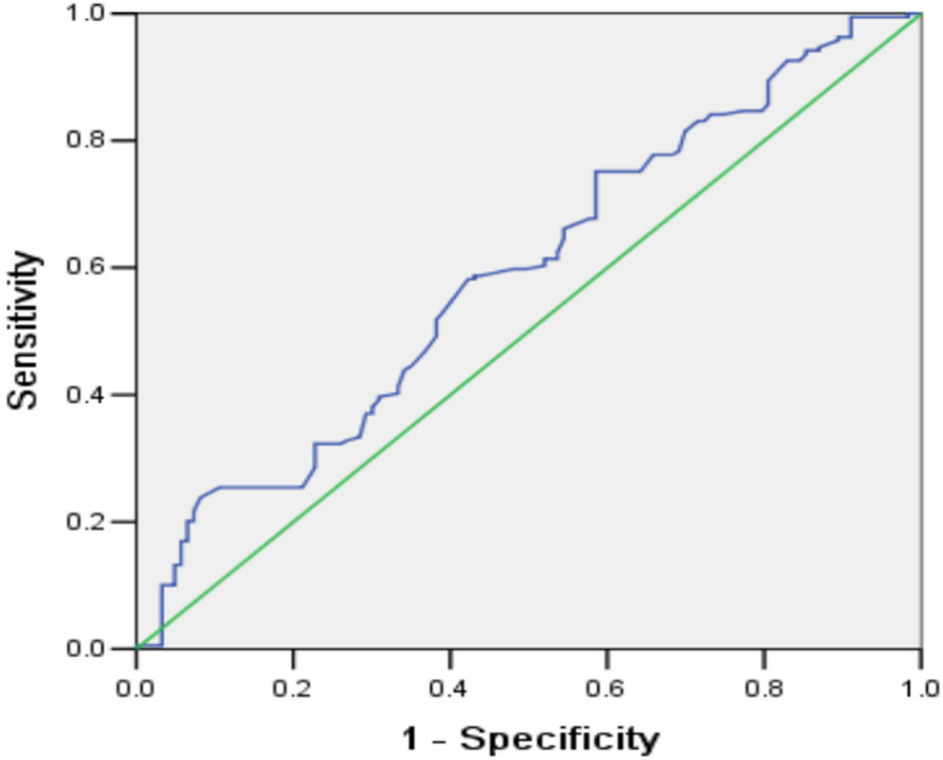
Receiver operating characteristic curve for the predictive value of NC for functional outcome in ICH patients at 6 months.

The univariate analysis showed that patients with poor functional outcome at 6 months had higher NC (*p* = 0.007), higher HNR (*p* = 0.015), higher hematoma size (*p* < 0.001), higher WBC count (*p* < 0.001), higher blood glucose level (*p* = 0.005), lower age (*p* = 0.036), and lower GCS score on admission (*p* < 0.001). The proportions of patients with IVH (31.7 vs. 50.8%, *p* = 0.001), endotracheal intubation (26.8 vs. 67.7%, *p* < 0.001), and tracheotomy (9.8 vs. 27.0%, *p* < 0.001), as well as patients with basal ganglia hemorrhage (40.7 vs. 55.0%) and brainstem hemorrhage (13.0 vs. 25.4%), were higher in the poor outcome group (Table 1). The multivariate analysis indicated that higher NC (OR = 1.205, 95% CI: 1.075–1.350, *p* = 0.001), lower BMI (OR = 0.829, 95% CI: 0.722–0.953, *p* = 0.009), GCS 9–12 points (OR = 4.139, 95% CI: 1.912–8.960, *p* < 0.001), GCS 3–8 points (OR = 57.537, 95% CI: 15.725–210.526, *p* < 0.001), larger hematoma size (OR = 1.062, 95% CI: 1.034–1.092, *p* < 0.001), basal ganglia hemorrhage (OR = 6.300, 95% CI: 2.285–17.375, *p* < 0.001), and brainstem hemorrhage (OR = 16.223, 95% CI: 3.226–81.572, *p* = 0.001) were significantly correlated with poor functional outcome at 6 months (Table 2). ROC curve analysis showed that NC could predict poor functional outcome with an area under the curve of 0.591 (95% CI: 0.527–0.655, *p* = 0.007, Figure 3). The value of 39.45 cm for NC was the best cut-off for predicting the functional outcome of ICH patients according to the Youden index, and the specificity, sensitivity, positive predictive value, and negative predictive value of NC for predicting poor outcome were 41.5, 71.5, 55, and 59.3%, respectively. In addition, we found that the proportions of poor outcomes in patients with NC ≥ 39.45 cm and patients with NC < 39.45 cm were 63.1 and 55.1%, respectively.

**Table 1:** Univariate analysis of clinical characteristics related to 180-day outcome in patients with ICH.

**Characteristic**	**Favorable outcome (*n* = 123)**	**Poor outcome (*n* = 189)**	***p*-value**
NC (cm)	41.01 ± 4.79	42.46 ± 4.68	0.007^*^
BMI (kg/m^2^)	25.46 ± 4.29	26.14 ± 3.75	0.142
HNR	4.03 ± 0.41	3.92 ± 0.37	0.015^*^
Age (years)	61 (49, 70)	56 (46, 67)	0.036^*^
Sex (male)	86 (69.9%)	143 (75.7%)	0.262
Hypertension	99 (80.5%)	164 (86.8%)	0.136
Diabetes mellitus	12 (9.8%)	9 (4.8%)	0.085
Prior stroke	3 (2.4%)	10 (5.3%)	0.260
Smoking	40 (32.5%)	52 (27.5%)	0.343
Alcohol consumption	26 (21.1%)	54 (28.6%)	0.142
SBP (mmHg)	161.70 ± 29.90	165.74 ± 27.98	0.207
DBP (mmHg)	94.58 ± 17.96	98.43 ± 17.74	0.063
GCS score on admission	–	–	<0.001^**^
13–15	80 (65.0%)	27 (14.3%)	–
9–12	38 (30.9%)	58 (30.7%)	–
3–8	5 (4.1%)	104 (55.0%)	–
ICH location	–	–	<0.001^**^
Lobe	32 (26.0%)	17 (9.0%)	–
Basal ganglia	50 (40.7%)	104 (55.0%)	–
Thalamus	18 (14.6%)	16 (8.5%)	–
Cerebellum	7 (5.7%)	4 (2.1%)	–
Brainstem	16 (13.0%)	48 (25.4%)	–
Hematoma size (ml)	15.0 (6.2, 27.3)	31.9 (15.3, 55.8)	<0.001^**^
Presence of IVH	39 (31.7%)	96 (50.8%)	0.001^*^
Endotracheal intubation	33 (26.8%)	128 (67.7%)	<0.001^**^
Tracheotomy	12 (9.8%)	51 (27.0%)	<0.001^**^
WBC, ×10^9^	9.98 ± 3.88	12.39 ± 4.09	<0.001^*^
Platelet, ×10^9^	173.98 ± 60.66	181.21 ± 69.63	0.347
Blood glucose (mmol/l)	7.75 ± 2.89	8.68 ± 2.75	0.005^*^
Cholesterol (mmol/l)	4.47 ± 1.08	4.43 ± 1.19	0.758
HDL-C (mmol/l)	1.35 ± 0.43	1.32 ± 0.52	0.568
LDL-C (mmol/l)	2.61 ± 0.77	2.59 ± 0.98	0.854

*Values are expressed as n (%), mean ± standard deviation, and median (25, 75%), as appropriate*.

* *p < 0.05*.

** *p < 0.001*.

*ICH, intracerebral hemorrhage; NC, neck circumference; BMI, body mass index; HNR, height-to-NC ratio; SBP, systolic blood pressure; DBP, diastolic blood pressure; GCS, Glasgow Coma Scale; IVH, intraventricular hemorrhage; WBC, white blood cell; HDL-C, high-density lipoprotein cholesterol; LDL-C, low-density lipoprotein cholesterol. Bold indicates p < 0.05*.

**Table 2:** Multivariate analysis of predictors for poor outcome at 6 months.

**Predictors**	**OR (95% CI)**	***p*-value**
NC (per 1 cm increase)	1.205 (1.075–1.350)	0.001^*^
BMI (per 1 kg/m^2^ increase)	0.829 (0.722–0.953)	0.009^*^
GCS score on admission	–	–
GCS (13–15 points)	Reference	–
GCS (9–12 points)	4.139 (1.912–8.960)	<0.001^**^
GCS (3–8 points)	57.537 (15.725–210.526)	<0.001^**^
ICH location	–	–
Lobe	Reference	–
Basal ganglia	6.300 (2.285–17.375)	<0.001^**^
Thalamus	3,751 (0.856–16.444)	0.080
Cerebellum	0.363 (0.045–2.893)	0.338
Brainstem	16.223 (3.226–81.572)	0.001^*^
Hematoma size (per 1 ml increase)	1.062 (1.034–1.092)	<0.001^**^
Presence of IVH	3.176 (1.476–6.834)	0.003^*^

*NC, neck circumference; BMI, body mass index; GCS, Glasgow Coma Scale; ICH, intracerebral hemorrhage; IVH, intraventricular hemorrhage. Bold indicates p < 0.05*.

* *p < 0.05*,

** *p < 0.001*.

## Discussion

To the best of our knowledge, this is the first study that focused on NC in patients with ICH. We found that NC was an independent risk factor for poor ICH prognosis, and that BMI was independently inversely associated with poor outcome in ICH patients. NC is an indicator of obesity that reflects health (16–21). Previous studies have shown that NC is associated with an increased risk of hypertension (18), diabetes (17, 21), and metabolic syndrome (19). NC is also found to be associated with congestive heart failure incidence and coronary heart disease mortality (20). However, research regarding the association between NC and ICH is limited.

According to the “obesity paradox,” obese and overweight stroke patients have a more favorable prognosis than those with a normal or lower BMI (22–24). Consistent with these findings, we found that BMI was independently inversely associated with poor outcome in ICH patients. Interestingly, we also found a contradictory relationship between BMI and NC in predicting the prognosis of ICH. One possible explanation for the paradoxical phenomenon may be that the two obesity indicators reflect different fat distributions. BMI only reflects total body obesity, and NC represents an alternative method for measuring upper body subcutaneous fat (25). Moreover, it has been demonstrated that NC is associated with oropharyngeal fatty infiltration, which narrows the upper airway, resulting in obstructive sleep apnea (OSA) (14).

A retrospective study found that among individuals with a larger NC (≥43.2 cm in men and ≥36.8 cm in women), the incidence of OSA was 2.52 times higher in men and 3.13 times higher in women (26). It was reported that compared with BMI, NC better explained the change in apnea–hypopnea index in morbidly obese women (*n* = 115) in the predictive model (27). OSA had been reported to be associated with ICH. The hypoxia and hemodynamic responses associated with OSA may predispose patients to stroke (28). Moreover, OSA was associated with the development of perihematoma edema (29), which may cause poor outcomes after ICH (30–32). Several mechanisms may influence the development of encephaledema after ICH. The early stage of cerebral edema occurs in the first few hours after ICH and involves hydrostatic pressure induced by the formation of hematoma and retraction of the clot. The second phase, caused by the production of thrombin and activation of the coagulation cascade, occurs within the first 24 h, and the delayed stage involves red blood cell hemolysis and hemoglobin-induced toxicity (33, 34). The sizes of perihematoma edema have also been related to several factors, such as the level of serum ferritin and increased matrix metalloproteinase-9 activity, which is an important enzyme for blood–brain barrier remodeling and perihematoma edema development (35–38).

Due to a momentary cessation of breathing, OSA patients with this disorder have repeated episodes of hypoxia/reoxygenation, promoting systemic oxidative stress, clotting cascade activation, inflammation, and damaged repair competence of the vascular endothelium (39). Thus, through the above several pathways, OSA may play a role in the generation of perihematoma edema. Moreover, OSA has been demonstrated to be associated with the enhanced activity of matrix metalloproteinase-9. Therefore, it is reasonable to believe that the association between OSA and perihematoma edema is biologically plausible.

Mechanical ventilation after tracheotomy or endotracheal intubation may prevent OSA-related hypoxia. However, a previous study reported that in acute spontaneous ICH patients, endotracheal intubation and mechanical ventilation were associated with an increased risk of hospital-acquired pneumonia and in-hospital mortality (40). Consistently, in the present study, we found that the proportions of patients with tracheotomy and endotracheal intubation were higher in the poor outcome group. The reason may be that the hematoma volume in ICH patients with tracheotomy or endotracheal intubation was larger than that in patients without tracheotomy or endotracheal intubation, which suggested a more severe condition and poorer prognosis.

In addition, previous studies have shown that NC was associated with an increased risk of hypertension (18), diabetes (17, 21), and metabolic syndrome (19), which also played a role in the occurrence and development of ICH (41–43). Furthermore, Pezzini et al. found that obesity, mainly through its indirect effect on hypertension and obesity-related complications, played a role in ICH (44). NC should not be overlooked in evaluating ICH patients.

Since NC was proven to predict poor outcome in patients with ICH, the potential clinical significance should be further studied. First, as an indicator of obesity, it is easy and convenient to obtain NC in clinical practice, especially among patients with poor coordination. Moreover, NC is related to oropharyngeal fat infiltration, which narrows the upper respiratory tract. For ICH patients with consciousness disturbance, NC can be used to evaluate endotracheal intubation or tracheotomy to keep the airway open. In addition, since a single predictor of outcome in ICH patients has limitations, several prognostic scores have been used to predict functional outcome and mortality in this population (45). Similarly, the combination of NC and other predictive factors can form new prediction scores with higher specificity and sensitivity. However, the potential clinical implications of NC should be further investigated.

However, our study has several limitations. First, this study collected data from one hospital, with a limited sample size, which may lead to selection bias. Second, NC is one of the factors causing upper respiratory tract stenosis, and the presence of other unmeasured factors will influence our final conclusion. Third, OSA was not accurately assessed in our study. Finally, all ICH patients were only from a West China Hospital, and patients with poor clinical conditions were usually recruited because of the medical referral system.

## Conclusion

NC is an independent predictor of unfavorable functional outcome at 6 months. Further experiments are necessary to explore the specific mechanism.

## Data Availability Statement

The raw data supporting the conclusions of this article will be made available by the authors, without undue reservation.

## Ethics Statement

The studies involving human participants were reviewed and approved by the ethics committee of West China Hospital of Sichuan University. The patients/participants provided their written informed consent to participate in this study.

## Author Contributions

YL and HL designed the project. YL performed the statistical analyses and wrote the manuscript draft. YL, HZ, FZ, JZ, XY, MX, and HL screened and extracted the data. All authors have made an intellectual contribution to the manuscript and approved the submission.

## Conflict of Interest

The authors declare that the research was conducted in the absence of any commercial or financial relationships that could be construed as a potential conflict of interest.
